# Enterovirus D68 in Viet Nam (2009-2015)

**DOI:** 10.12688/wellcomeopenres.11558.2

**Published:** 2018-05-11

**Authors:** Nguyen Thi Han Ny, Nguyen To Anh, Vu Thi Ty Hang, Lam Anh Nguyet, Tran Tan Thanh, Do Quang Ha, Ngo Ngoc Quang Minh, Do Lien Anh Ha, Angela McBride, Ha Manh Tuan, Stephen Baker, Pham Thi Thanh Tam, Tran My Phuc, Dang Thao Huong, Tran Quoc Loi, Nguyen Tran Anh Vu, Nguyen Van Hung, Tran Thi Thuy Minh, Nguyen Van Xang, Nguyen Dong, Ho Dang Trung Nghia, Nguyen Van Vinh Chau, Guy Thwaites, H. Rogier van Doorn, Catherine Anscombe, Tan Le Van, VIZIONS Consortium

**Affiliations:** 1 Oxford University Clinical Research Unit, Ho Chi Minh City, Vietnam; 2 Ho Chi Minh City University of Science, Ho Chi Minh City, Vietnam; 3 Children's Hospital 1, Ho Chi Minh City, Vietnam; 4 Murdoch Children's Research Institute, Melbourne, Australia; 5 Children's Hospital 2, Ho Chi Minh City, Vietnam; 6 Centre for Tropical Medicine, Nuffield Department of Medicine, University of Oxford, Oxford, UK; 7 Dong Thap General Hospital, Ban Me Thuot City, Vietnam; 8 Dak Lak General Hospital, Ban Me Thuot City, Vietnam; 9 Khanh Hoa General Hospital, Nha Trang City, Vietnam; 10 Pham Ngoc Thach University, Ho Chi Minh City, Vietnam; 11 Hospital for Tropical Diseases, Ho Chi Minh City, Vietnam

**Keywords:** Enterovirus D68, respiratory infections, VIZIONS, next generation sequencing, Vietnam

## Abstract

**Background: **Since 1962, enterovirus D68 (EV-D68) has been implicated in multiple outbreaks and sporadic cases of respiratory infection worldwide, especially in the USA and Europe with an increasing frequency between 2010 and 2014. We describe the detection, associated clinical features and molecular characterization of EV-D68 in central and southern Viet Nam between 2009 and 2015.

**Methods:** Enterovirus/rhinovirus PCR positive respiratory or CSF samples taken from children and adults with respiratory/central nervous system infections in Viet Nam were tested by an EV-D68 specific PCR. The included samples were derived from 3 different observational studies conducted at referral hospitals across central and southern Viet Nam 2009  2015. Whole-genome sequencing was carried out using a MiSeq based approach. Phylogenetic reconstruction and estimation of evolutionary rate and recombination were carried out in BEAST and Recombination Detection Program, respectively.

**Results:** EV-D68 was detected in 21/625 (3.4%) enterovirus/rhinovirus PCR positive respiratory samples but in none of the 15 CSF. All the EV-D68 patients were young children (age range: 11.8 – 24.5 months) and had moderate respiratory infections. Phylogenetic analysis suggested that the Vietnamese sequences clustered with those from Asian countries, of which 9 fell in the B1 clade, and the remaining sequence was identified within the A2 clade. One intra sub-clade recombination event was detected, representing the second reported recombination within EV-D68. The evolutionary rate of EV-D68 was estimated to be 5.12E ^-3 ^substitutions/site/year. Phylogenetic analysis indicated that the virus was imported into Viet Nam in 2008.

**Conclusions:** We have demonstrated for the first time EV-D68 has been circulating at low levels in Viet Nam since 2008, associated with moderate acute respiratory infection in children. EV-D68 in Viet Nam is most closely related to Asian viruses, and clusters separately from recent US and European viruses that were suggested to be associated with acute flaccid paralysis.

## Introduction

Enterovirus D68 (EV-D68) is a genotype of Enterovirus D, a species within the genus Enterovirus, family *Picornaviridae*. It was initially isolated in 1962 from children with bronchitis/pneumonia in California, USA ^1^. EV-D68 shares numerous properties with rhinoviruses, including its association with respiratory rather than systemic infections and, unlike other enterovirus A–D genotypes, its 5’ untranslated region (5’ UTR) end ^2, 3^. EV-D68 can be further divided into clades A, B and C and these can be further divided into subclades (e.g A1, A2).

Since 1962, EV-D68 has been implicated in multiple small outbreaks and sporadic cases of respiratory infection worldwide, with the associated clinical syndromes ranging in severity from mild to severe. However, between 2010 and 2014 EV-D68 was reported to the Centres of Disease Control and Prevention in the USA (USCDC) at much higher frequency than in previous years: 1153 cases were confirmed, predominantly among children and often in a context of asthma and wheezing. A simultaneous increase in numbers of cases of acute flaccid paralysis was reported, and although an epidemiological link seems possible, a virological association between these events has not yet been proven ^4, 5^. Genomic investigation of the causative viruses of those outbreaks in the USA showed that the EV-D68 belonged to the subclade B1 ^4^. Increased detections of EV-D68 were also reported in both children and adults from Europe and the Asia-Pacific region during the 2014–2015 period ^6– 10^.

Given the emergence and potential public health threat of EV-D68, improving our knowledge about the geographic distribution, evolution and associated clinical phenotypes of the virus is essential for future intervention strategies and outbreak response. Here we describe the detection, associated clinical features and molecular characterization of EV-D68 infection, using respiratory and cerebrospinal fluid (CSF) samples from children in central and southern Viet Nam between 2009 and 2015.

## Methods

### Clinical samples

Clinical samples were selected from three different studies previously conducted in Viet Nam. The first cohort involved children under two years of age with lower respiratory tract infections admitted to two large paediatric hospitals (Children’s Hospital 1 and 2, Ho Chi Minh City) between 2009 and 2010 [n=632, median age: 7 months, interquartile range (IQR): 4 – 12] ^11^. The second cohort involved children with respiratory infections visiting the outpatient department of Children’s Hospital 1 between 2009 and 2010 (n=563; median age: 1.96 years, IQR: 1.05 – 3.18) ^12^. The third cohort involved adults and children hospitalised with respiratory or central nervous system infections, admitted to five major hospitals in central and southern Viet Nam between 2013 and 2016 ^13^, with respiratory samples (n= 3791, median age: 2 years, IQR: 1 – 4) and CSF samples (n= 877, median age: 17 years, IQR: 5 – 44) being taken. The five hospitals in Viet Nam included: i) Dong Thap General Hospital, Dong Thap province, ii) Hue Central Hospital, Hue City, iii) Dak Lak General Hospital, Ban Me Thuot City, iv) Khanh Hoa General Hospital, Nha Trang City, and v) Hospital for Tropical Diseases, Ho Chi Minh City.

### Ethics

All studies were approved by the corresponding institutional review board of the local hospitals in Viet Nam where patients were enrolled:

(i) Children Hospital 1, Ho Chi Minh City (approval numbers 430BVNĐ and 146/BVNĐ1-KHKT);
(ii) Hue Central Hospital, Hue City (77/25/05/12);
(iii) Dak Lak General Hospital, Ban Me Thuot City (489/BVT-KHTH);
(iv) Khanh Hoa General Hospital, Nha Trang City (356/BVĐKT);
(v) Hospital for Tropical Diseases, Ho Chi Minh City (136/BVBNĐ – KH);
(vi) Children Hospital 2, Ho Chi Minh City,
(vii) Dong Thap General Hospital, Dong Thap province (approval number not available; signed date: 5/6/12.

The study was also approved by the Oxford Tropical Research Ethics Committee (31-08, 44-08 and 15-12).

Written informed consent was obtained from either the participant, or the participant’s parent or legal guardian.

### Enterovirus D68 detection and whole genome sequencing

From the above described cohorts, archived respiratory samples and CSF were screened using a 5’ UTR PCR ^14^, those that were enterovirus or rhinovirus PCR positive were selected for further testing by EV-D68 viral specific PCR ^15^. EV-D68 real time specific reverse-transcriptase PCR (RT-PCR) was performed using D68 AN887 primers, D68 AN890 probe and SuperScrip III One-Step RT-PCR system with Platinum Taq (Invitrogen, Carlsbad, CA, USA). PCR amplification was carried out as described in the original publication ^15^. In brief, in a total reaction volume of 20 μl, the PCR mixture consisted of 5 μl of template RNA, 0.6 μM of primers D68 AN887 and 0.8 μM probe D68 AN890, 10 μl of Platinum PCR supermix (Invitrogen) and 0.4 μl enzyme SuperScript III One-Step RT-PCR. The thermal cycling condition consisted of 1 cycle of 50°C for 30 min, 1 cycle of 95°C for 2 min followed by 45 cycles of 95°C for 15sec, 55°C for 1 min and 72°C for 10sec. All RT-PCR reactions were performed in a LightCycler480 II (Roche Diagnostics, Mannheim, Germany).

EV-D68 positive specimens with a Ct-value of 32 or less were whole-genome sequenced using an in-house non-ribosomal random PCR and MiSeq based approach ^16, 17^. All the experiments were carried out as previously described ^17^. In short, extracted viral nucleic acids from nuclease-treated clinical samples were randomly amplified using non-ribosomal random PCR. The resulting PCR products were quantified by QIAquick PCR purification kit (QIAgen GmbH, Hilden, Germany) and measured by Qubit dsDNA HS kit (Invitrogen). One nanogram of the purified DNA was then subjected to library preparation using the Nextera XT DNA sample preparation kit (Illumina, San Diego, CA, USA), each sample was allocated to a barcode sequence using the Nextera XT Index Kit (Illumina). Sequencing of the prepared library was carried out using the MiSeq reagent kit V3 in an Illumina Miseq platform (Illumina). A total of 96 samples were sequenced in a single run.

### Sequence analysis

The generated sequencing data from Illumina MiSeq was first subjected to a primer removing step using standard parameters available in Geneious software version 8.1.5 (Biomatters, Ltd, Auckland, New Zealand) ^17, 18^. A reference based mapping approach was then employed to assemble the viral genomes, followed by manual editing of the obtained consensuses using Geneious. Samples where the full VP1 regions or whole genomes were successfully sequenced proceeded to recombination detection and phylogenetic analysis.

### Recombination detection and phylogenetic analysis

All sequence alignment was carried out using MUSCLE, available in Geneious (Biomatters).

Recombination was inferred using a combination of methods (Chimera, GENECONV, Maxchi, Bootscan and Siscan) within RDP4 (Recombination Detection Program, version 4) ^19^, with recombination supported if more than three methods showed significant values. The recombination event was then confirmed by constructing a neighbor-joining tree using the group D enterovirus sequences. Identified recombined samples were removed from further phylogenetic analysis.

The origin, evolution rate and divergence time of EV-D68 were estimated by using representatives of VP1 sequences (n=124) and whole genome sequences (n=58), downloaded from GenBank ( Supplementary Table 1 and Supplementary Table 2) alongside the Vietnamese sequences recovered in the present study. All analyses were carried out in BEAST version 1.8.3 ^20^ using the General Time Reversible (GTR) with gamma 4 nucleotide substitution model and the strict molecular clock model and support for individual nodes was assessed using a bootstrap produce (1000 replicates). The molecular model was selected using Bayes factor. The Bayesian MCMC chain lengths were 100 million generations with sampling every 1000 generations.

### Sequence accession numbers

The sequences of EV-D68 obtained in this study were submitted to NCBI under accession numbers MF045413–MF045423.

## Results

Of a total 5863 samples (4986 respiratory samples and 877 CSF), 639 (624 respiratory, 15 CSF) were positive for enterovirus or rhinovirus by PCR on initial screening. EV-D68 was subsequently detected in 21 of 624 (3.4%) of enterovirus/ rhinovirus positive respiratory samples, while no CSF samples were positive for EV-D68. Overall, EV-D68 was detected in 0.4% of 5863 tested samples, and 3.3% of 639 enterovirus/ rhinovirus positive respiratory/CSF samples. The earlier EV-D68 PCR positive sample was collected on the 4th December 2009.

### Demographics and clinical features

Table 1 briefly summarises the demographics, presenting features and outcomes for all 21 EV-D68 PCR positive patients. All 21 patients in whom EV-D68 was detected were aged 2 years or less, with a median age of 17 months at time of presentation. Three were admitted to the outpatient department of Children’s Hospital 1 (the second cohort), and the remaining 18 were inpatients enrolled into the first and the third cohorts. One patient had a pre-existing neurological comorbidity; the remaining 20 children were healthy at baseline.

**Table 1.** Demographics, clinical features and outcome for 21 patients with EV-D68 PCR positive respiratory specimens. Categorical data were presented as n (%). Continuous variables were presented as median (range).

Characteristics	
**Male**	13 (61.9)
**Female**	8 (38.1)
**Age (months)**	17.2 (11.8-24.5)
**Clinical features**	
**Acute respiratory illness**	21 (100)
**Duration of symptoms prior to admission** **(days)**	2 (2-3)
**Fever**	13 (61.9)
**Temperature at presentation**	38.0 (37.5-38.4)
**Haematology results**	
**Haemoglobin (g/dL)**	11.2 (10.3-12.0)
**Leucocyte count ×10 ^9^/L**	13.6 (10.9-16.5)
**Neutrophils % total**	57.3 (37.8-64.9)
**Lymphocytes % total**	30.45 (22.5-43)
**Eosinophils % total**	0.25 (0.2-1.9)
**Platelets**	347 (316-377)
Discharge outcome*	
**Complete recovery**	16 (76.2)
**Residual symptoms**	5 (23.8)
**Death**	0 (0)

All 21 patients presented with an acute respiratory illness of short duration (median onset 2 days prior to admission), and the median temperature at presentation was 38°C (range: 37.5°C – 38.4°C) ( Table 1). No patient required admission to intensive care. All of the patients survived their infection, and 16 (76%) had recovered completely at the time of discharge. The nature of the residual symptoms in the remaining 5 patients was not available. There were no reported cases of acute flaccid paralysis in this case series.

### Whole genome sequencing

In total, 15 samples were processed for whole genome sequencing; of these, 9 samples gave genome coverage of over 89%, with the uncovered sections being mainly confined to the 5’ end of the genome. Two additional samples gave complete coverage of the VP1 region.

### Recombination detection

**Figure 1.**
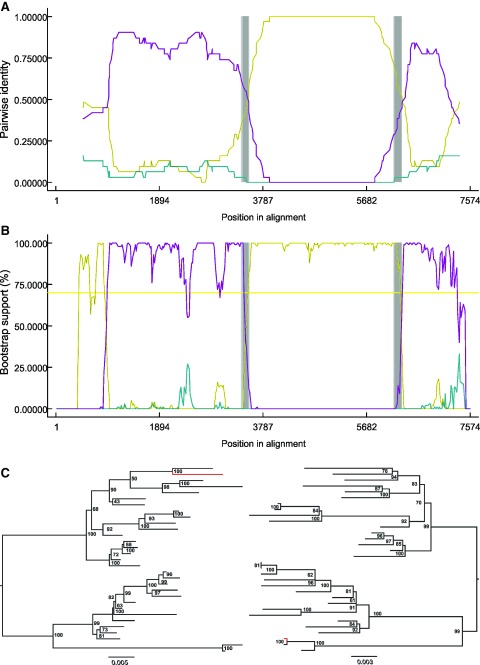
Recombination analysis of EVD68-VN9. ( **A**) Similarity plot analysis of EVD68-VN5 against the two heterogenic parents, EVD68-VN3 (blue) and EVD68-VN7 (purple). The x-axis indicates the number of nucleotides along the alignment, the y-axis indicates percentage similarity. ( **B**) Bootscan plot analysis supporting for the recombination event of EVD68-VN5 detected by similarity plot analysis in ( **A**). The y-axis indicates the percentage of bootstrap values. ( **C**) Phylogenetic analyses of nonrecombinant fragments of representatives of clade B1; the putative recombinant strain is in red.

One recombination event was detected, in sample EVD68-VN5, with the recombination being intra sub-clade, within B1 ( Figure 1). The recombination event occurred between nucleotide 3500 and 6300, with the sequence assembly depth remaining at about 1000 across the whole genome and phylogenetic tree, with a change in topological position within the B1 clade supported by the recombination event ( Figure 1C). Sample EVD68-VN5 was therefore removed from subsequent phylogenetic inference.

### Phylogenetic analysis and evolutionary rate of EV-D68

**Figure 2.**
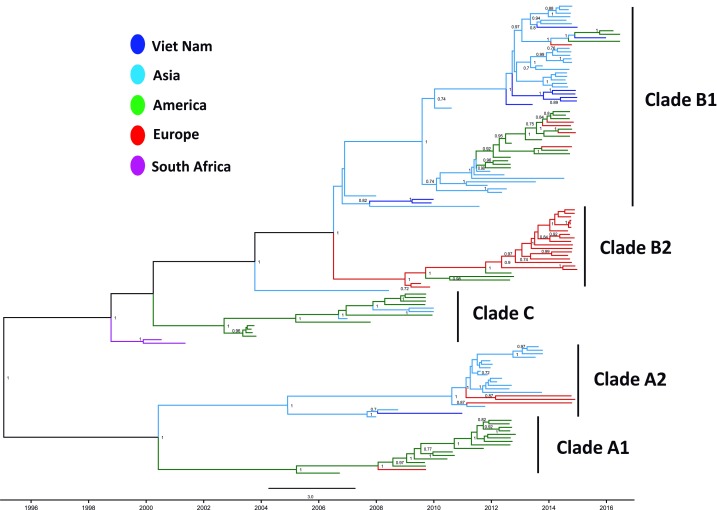
MCC tree from Bayesian timescale phylogenetic analysis based on complete VP1 nucleotide sequence (927nt) of EV-D68 including Vietnamese strains obtained from this study (dark blue) and global representatives retrieved from GenBank.

**Figure 3.**
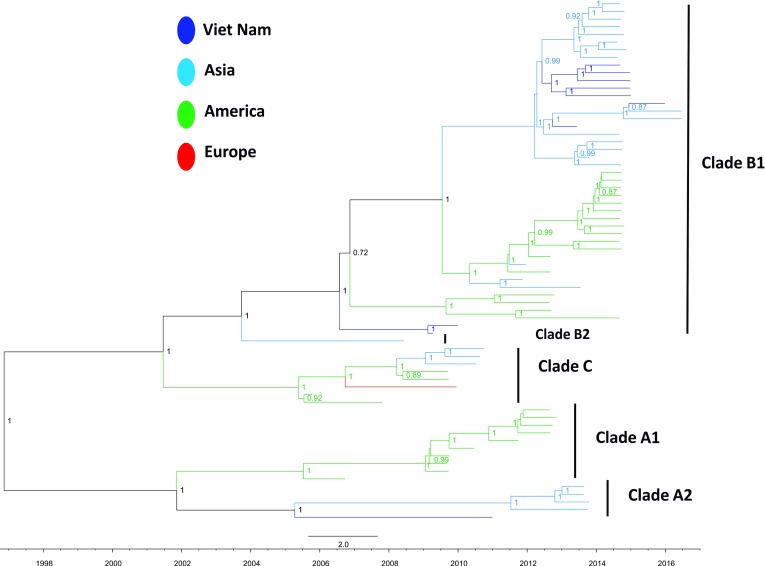
MCC tree from Bayesian timescale phylogenetic analysis based on complete genomes of EV-D68 including Vietnamese strains obtained from this study (dark blue) and global representatives retrieved from GenBank.

9 out of 10 Vietnamese EV-D68 VP1 sequences fell in the B1 clade ( Figure 2), and the remaining sequence was identified within the A2 clade. In all cases, the Vietnamese sequences clustered with Asian viruses. Whilst the Vietnamese viruses were not monophyletic, seven of the B1 sequences had other Vietnamese sequences as their closest relatives. Phylogenetic analysis also suggests that the analysis was carried out at whole genome level ( Figure 3), with the exception that a single sequence from the A2 clade was introduced earlier (2006).

The rate of evolution of EV-D68 was calculated from the VP1 data as 5.12E ^-3^ substitutions/site/year. Bayesian analysis suggests that the EV-D68 origin lies in late 1960, and the common ancestor of the lineages under-investigation arose in 1994.

## Discussion

Herein we have described for the first time the clinical presentation and phylogenetic characterization of EV-D68 in Viet Nam for the period 2009 to 2015.

Of 639 patients whose nasopharyngeal or CSF sample tested positive for enterovirus or rhinovirus on multiplex PCR, 21 (3.2%) respiratory samples were found to be positive for EV-D68 on specific RT-PCR. Overall, of 4986 respiratory samples screened, 0.4% were positive for EV-D68. This indicates that EV-D68 has been circulating at low levels in Viet Nam between 2009 and 2015; however, it does not represent a major contribution to the burden of acute respiratory illness in the region, in line with findings by Nguyen *et al.* that show enteroviruses only account for 5% of respiratory illness in Viet Nam ^21^. The EV-D68 specific RT-PCR used in the present study has been shown to have a sensitivity and specificity of 98.6% and 97.5% respectively ^15^, however evaluation of assay performance has only taken place in the United States. Therefore, the assay sensitivity and specificity on South-Easrt Asian clades of EV-D68 remain unknown and thus some may have been missed in our cohort, although as originally designed, the assay should be able to detect all EV-D68 strains from Asia, Europe and the US.

All of 21 EV-D68 positive patients were aged less than 2 years, presented with a respiratory illness of short duration and none needed admission to intensive care units. This is consistent with the findings of Xiang *et al.* ^22^ who reported that EV-D68 infection predominantly caused non-severe respiratory illness in children in China between 2006 and 2014, but differs from reports from Thailand and Cambodia, where EV-D68 positive patients were mostly older children and adults, respectively ^23, 24^. It should be noted, however, that the majority of our patients were children, which may explain the difference. Likewise, it has recently been reported in the Netherlands that EV-D68 was associated with severe respiratory infection in young children, however most of these patients had pulmonary co-morbidity at baseline ^25^.

Our phylogenetic analysis puts the identified Vietnamese EV-D68 in a global context and shows that the EV-D68 viruses circulating in Viet Nam belong to subclade B1, which includes EV-D68 viruses sampled across various continents including Asia, Europe and America. Our Vietnamese viruses are, however, most closely related to other Asian strains, and cluster separately within subgroup B1 from those which have been associated with epidemic outbreaks and implicated in acute flaccid paralysis in the USA in children with a median age of 8 years ^4^. It should however be noted that to date there has been no definitive link between specific EV-D68 strains and clinical phenotypes. Likewise, the causative role of EV-D68 in acute flaccid paralysis remains unproven.

Interestingly, we report herein a recombination event within our EV-D68 whole genome data set. This represents the second reported recombination within EV-D68 ^26^, although recombination is a common phenomenon of enterovirus evolution.

The non-monophyletic clustering pattern suggests that EV-D68 was introduced in Viet Nam multiple times, while the low-level clustering suggests some persistence within Viet Nam, with no outbreak reported until now. Our data agrees with other published estimations of the origin and evolution rate of EV-D68 ^23, 27^.

## Conclusion

We have demonstrated that EV-D68 has been circulating at low levels in Viet Nam in the period of 2009 to 2015, and is associated with a moderate acute respiratory infection in healthy children in our cohorts. EV-D68 in Viet Nam is most closely related to other circulating Asian strains, and clusters separately from those implicated to be associated with acute flaccid paralysis in the USA and Europe.

## Data availability

The sequences of EV-D68 obtained in this study were submitted to NCBI under accession numbers MF045413–MF045423.

## Supplementary materials

**Supplementary Table 1: Accession numbers, locations and sampling dates of representatives of VP1 sequences.**

**Supplementary Table 2: Accession numbers, locations and sampling dates of representatives of whole genome sequences used for the analysis.**
