# Supplementary material for: Enterovirus D68 in Viet Nam (2009-2015)
Source: Wellcome Open Res. 2018 May 11;2:41. Originally published 2017 Jun 15. [Version 2] doi: 10.12688/wellcomeopenres.11558.2 (PMC5553084; doi:10.12688/wellcomeopenres.11558.2)
Supplement: Supplementary file 1 [file wellcomeopenres-2-15891-s0000.tgz › 3e660984-a3d2-4e08-91b9-42c0d2ffe6c6.pdf]

Supplementary Table 1: Accession numbers, locations and sampling dates of representatives of VP1 sequences used for the analysis

No	Accession number/location/sampling date
1	AB614428/Japan/EVD68/2010
2	AB614439/Japan/EVD68/2010
3	AB667895/Japan/EVD68/2007
4	AB667899/Japan/EVD68/2008
5	AB861414/Philippines/EVD68/2011-08-04
6	JX070222/New-Zealand/EVD68/2010-06-23
7	JX101795/South-Africa/EVD68/2001-05
8	JX101799/South-Africa/EVD68/2000-07
9	KF726085/China/EVD68/2008-10-06
10	KP114663/Canada/EVD68/2014-08-31
11	KP114665/Canada/EVD68/2014-09-02
12	KP153540/Italy/EVD68/2014-10-20
13	KP189395/Germany/EVD68/2014-10-09
14	KP189399/Germany/EVD68/2014-10-13
15	KP189401/Germany/EVD68/2014-10-23
16	KP189403/Germany/EVD68/2014-10-20
17	KP307991/France/EVD68/2014-11-12
18	KP307992/France/EVD68/2014-11-23
19	KP406489/France/EVD68/2014-10-10
20	KP406490/France/EVD68/2014-10-11
21	KP657737/Germany/EVD68/2014-09-01
22	KP657741/Germany/EVD68/2014-10-07
23	KP657743/Germany/EVD68/2014-10-13
24	KP657744/Germany/EVD68/2014-10-16
25	KP657745/Germany/EVD68/2014-10-20
26	KP657746/Germany/EVD68/2014-10-27
27	KP745762/USA/EVD68/2014-09-23
28	KP745766/USA/EVD68/2014-09-26
29	KP745767/USA/EVD68/2014-09-26
30	KP745769/USA/EVD68/2014-09-25
31	KT220448/France/EVD68/2014-12-02
32	KT220463/France/EVD68/2014-09-27
33	KT220478/France/EVD68/2014-11-25
34	KT220486/France/EVD68/2014-11-27
35	KT220490/France/EVD68/2014-12-03
36	KT220492/France/EVD68/2014-12-09
37	KT220499/France/EVD68/2014-10-21
38	KT220501/France/EVD68/2014-11-02

39	KT220505/France/EVD68/2014-12-28
40	KT231898/Netherlands/EVD68/2009-07-24
41	KT231900/Netherlands/EVD68/2009-09-25
42	KT231902/Netherlands/EVD68/2009-11-16
43	KT231904/Netherlands/EVD68/2009-12-15
44	KT231906/Netherlands/EVD68/2010-08-30
45	KT231908/Netherlands/EVD68/2010-09-24
46	KT280497/China/EVD68/2014-10-20
47	KT280499/China/EVD68/2014-10-15
48	KT280501/China/EVD68/2013-10-17
49	KT280502/China/EVD68/2013-08-25
50	KT280503/China/EVD68/2011-11-15
51	KT280504/China/EVD68/2013-07-20
52	KT285319/China/EVD68/2012-01-18
53	KT285320/China/EVD68/2011-12-19
54	KT285480/China/EVD68/2011-12-23
55	KT285484/China/EVD68/2008-06-12
56	KT306743/China/EVD68/2011-12-20
57	KT318494/Taiwan/EVD68/2008
58	KT347280/USA/EVD68/2012-11-07
59	KT711078/Taiwan/EVD68/2014-08-19
60	KT711079/Taiwan/EVD68/2014-08-22
61	KT711080/Taiwan/EVD68/2014-09-02
62	KT711082/Taiwan/EVD68/2014-08-20
63	KT711084/Taiwan/EVD68/2014-08-01
64	KT711086/Taiwan/EVD68/2014-08-12
65	KT711087/Taiwan/EVD68/2014-09-11
66	KT803588/China/EVD68/2013-10-05
67	KT803590/China/EVD68/2013-10-16
68	KT803591/China/EVD68/2014-09-18
69	KT803593/China/EVD68/2014-09-22
70	KT803595/China/EVD68/2014-10-13
71	KT803597/China/EVD68/2014-09-30
72	KT803600/China/EVD68/2014-10-25
73	KT803604/China/EVD68/2014-09-30
74	KT803605/China/EVD68/2014-10-07
75	KT803606/China/EVD68/2014-10-22
76	KT825142/Mexico/EVD68/2014-10-23
77	KT959176/Hong-Kong/EVD68/2010-08
78	KT959177/Hong-Kong/EVD68/2011-08
79	KT959178/Hong-Kong/EVD68/2011-10

80	KT959179/Hong-Kong/EVD68/2011-11
81	KT959183/Hong-Kong/EVD68/2012-03
82	KT959184/Hong-Kong/EVD68/2012-05
83	KT959185/Hong-Kong/EVD68/2012-05
84	KT959186/Hong-Kong/EVD68/2012-06
85	KT959188/Hong-Kong/EVD68/2012-07
86	KT959189/Hong-Kong/EVD68/2012-08
87	KT959190/Hong-Kong/EVD68/2012-09
88	KT959193/Hong-Kong/EVD68/2013-07
89	KT959196/Hong-Kong/EVD68/2013-10
90	KT959198/Hong-Kong/EVD68/2014-07
91	KX255355/USA/EVD68/2009-09-11
92	KX255359/USA/EVD68/2012-09-14
93	KX255361/USA/EVD68/2012-09-27
94	KX255363/USA/EVD68/2006-09-26
95	KX255365/USA/EVD68/2012-09-05
96	KX255369/USA/EVD68/2012-08-21
97	KX255372/USA/EVD68/2003-11-03
98	KX255375/USA/EVD68/2014-09-26
99	KX255376/USA/EVD68/2011-09-27
100	KX255377/USA/EVD68/2012-09-03
101	KX255380/USA/EVD68/2012-08-29
102	KX255381/USA/EVD68/2012-09-04
103	KX255382/USA/EVD68/2014-08-05
104	KX255383/USA/EVD68/2012-09-04
105	KX255386/USA/EVD68/2012-08-31
106	KX255387/USA/EVD68/2013-10-07
107	KX255388/USA/EVD68/2003-09-02
108	KX255394/USA/EVD68/2012-09-14
109	KX255397/USA/EVD68/2007-10-22
110	KX255399/USA/EVD68/2012-08-02
111	KX255400/USA/EVD68/2003-10-09
112	KX255401/USA/EVD68/2012-08-27
113	KX255404/USA/EVD68/2012-09-21
114	KX255408/USA/EVD68/2009-09-15
115	KX255410/USA/EVD68/2003-09-17
116	KX261821/USA/EVD68/2009-09-24
117	KX261823/USA/EVD68/2009-09-21
118	KX261824/USA/EVD68/2009-09-23
119	KX261826/USA/EVD68/2009-09-27
120	KX433158/USA/EVD68/2012-09-17

121	KX433164/USA/EVD68/2012-10-16
122	KX675261/USA/EVD68/2016-03-31
123	KX675262/USA/EVD68/2016-06-24
124	KX675263/USA/EVD68/2016-06-24
