# Supplementary material for: Enterovirus D68 in Viet Nam (2009-2015)
Source: Wellcome Open Res. 2018 May 11;2:41. Originally published 2017 Jun 15. [Version 2] doi: 10.12688/wellcomeopenres.11558.2 (PMC5553084; doi:10.12688/wellcomeopenres.11558.2)
Supplement: Supplementary file 2 [file wellcomeopenres-2-15891-s0001.tgz › 87c1b039-ea50-4349-ae82-a9bdcadc3306.pdf]

Supplementary Table 2: Accession numbers, locations and sampling dates of representatives of whole genome sequences used for the analysis

No	accession numbers/location/samplingdate
1	AB601882/Japan/EVD68/2010
2	AB601884/Japan/EVD68/2010
3	AB601885/Japan/EVD68/2010
4	JX070222/New-Zealand/EVD68/2010-06-23
5	KP114663/Canada/EVD68/2014-08-31
6	KP114665/Canada/EVD68/2014-09-02
7	KP745753/USA/EVD68/2014-09-28
8	KP745760/USA/EVD68/2014-09-19
9	KP745761/USA/EVD68/2014-09-22
10	KP745762/USA/EVD68/2014-09-23
11	KP745765/USA/EVD68/2014-09-25
12	KP745767/USA/EVD68/2014-09-26
13	KT231903/Netherlands/EVD68/2009-12-15
14	KT280496/China/EVD68/2014-11-19
15	KT280497/China/EVD68/2014-10-20
16	KT280499/China/EVD68/2014-10-15
17	KT280501/China/EVD68/2013-10-17
18	KT280502/China/EVD68/2013-08-25
19	KT280503/China/EVD68/2011-11-15
20	KT280504/China/EVD68/2013-07-20
21	KT285320/China/EVD68/2011-12-19
22	KT285484/China/EVD68/2008-06-12
23	KT285485/China/EVD68/2014-08-11
24	KT347280/USA/EVD68/2012-11-07
25	KT711080/Taiwan/EVD68/2014-09-02
26	KT711086/Taiwan/EVD68/2014-08-12
27	KT711087/Taiwan/EVD68/2014-09-11
28	KT764078/China/EVD68/2013-08-22
29	KT803588/China/EVD68/2013-10-05
30	KT803590/China/EVD68/2013-10-16
31	KT803591/China/EVD68/2014-09-18
32	KT803592/China/EVD68/2014-09-11
33	KT803597/China/EVD68/2014-09-30
34	KT803598/China/EVD68/2014-09-18
35	KT803600/China/EVD68/2014-10-25
36	KT825142/Mexico/EVD68/2014-10-23
37	KX255361/USA/EVD68/2012-09-27
38	KX255363/USA/EVD68/2006-09-26

39	KX255365/USA/EVD68/2012-09-05
40	KX255367/USA/EVD68/2014-09-12
41	KX255375/USA/EVD68/2014-09-26
42	KX255376/USA/EVD68/2011-09-27
43	KX255381/USA/EVD68/2012-09-04
44	KX255383/USA/EVD68/2012-09-04
45	KX255386/USA/EVD68/2012-08-31
46	KX255389/USA/EVD68/2014-09-26
47	KX255393/USA/EVD68/2005-10-17
48	KX255394/USA/EVD68/2012-09-14
49	KX255397/USA/EVD68/2007-10-22
50	KX255401/USA/EVD68/2012-08-27
51	KX255408/USA/EVD68/2009-09-15
52	KX255409/USA/EVD68/2009-09-04
53	KX261804/USA/EVD68/2009-10-05
54	KX261821/USA/EVD68/2009-09-24
55	KX261824/USA/EVD68/2009-09-23
56	KX433164/USA/EVD68/2012-10-16
57	KX675262/USA/EVD68/2016-06-24
58	KX675263/USA/EVD68/2016-06-24
