# Supplementary material for: Enterovirus D68 in Viet Nam (2009-2015)
Source: Wellcome Open Res. 2018 May 11;2:41. Originally published 2017 Jun 15. [Version 2] doi: 10.12688/wellcomeopenres.11558.2 (PMC5553084; doi:10.12688/wellcomeopenres.11558.2)
Supplement: Peer review history

# Peer review: Referee response for version 2

**DOI:** 10.21956/wellcomeopenres.15891.r33134

**Published:** 2018-05-11

**Authors:** Weihua Huang

**Affiliations:** 1 Department of Pathology, New York Medical College, Vahalla, NY, USA

Approved. No further comments to make. Thanks.

I have read this submission. I believe that I have an appropriate level of expertise to confirm that it is of an acceptable scientific standard.

# Peer review: Referee response for version 1

**DOI:** 10.21956/wellcomeopenres.12486.r24362

**Published:** 2017-08-07

**Authors:** Hitoshi Oshitani

**Affiliations:** 1 Department of Virology, Tohoku University Graduate School of Medicine, Sendai, Japan

The manuscript is well written and technically sound. But I have a few minor comments which might be helpful to improve the manuscript. 

1. In the introduction, the authors mentioned that EV-D68 is a genotype. It is true that typing of enteroviruses including EV-D68 is currently done mainly by genetic analysis. But many of enteroviruses including EV-D68 were originally classified as serotypes by using serological tests. I don’t think a genotype is appropriate.
2. In line 7 of introduction, I am not sure what the authors would like to explain about 5’UTR.
3. They mentioned that all EV-D68 cases had ‘moderate respiratory illness’. But the definition of moderate respiratory illness is unclear.
4. They collected samples from 2009 and 2015. But it is not mentioned in which year EV-D68 were detected.

I have read this submission. I believe that I have an appropriate level of expertise to confirm that it is of an acceptable scientific standard.

# Response

**Published:** 2018-05-03

**Authors:** Nguyen Thi Han Ny

**Affiliations:** Oxford University Clinical Research Unit, Vietnam

The manuscript is well written and technically sound. But I have a few minor comments which might be helpful to improve the manuscript. 

- In the introduction, the authors mentioned that EV-D68 is a genotype. It is true that typing of enteroviruses including EV-D68 is currently done mainly by genetic analysis. But many of enteroviruses including EV-D68 were originally classified as serotypes by using serological tests. I don’t think a genotype is appropriate.

**Response**: Thank you for your comment. We have updated the paper to use serotype to describe EV-D68 throughout.

 

- In line 7 of introduction, I am not sure what the authors would like to explain about 5’UTR.

**Response: **A sentence describing the 5’ UTR has been updated to make our point clearer.

- They mentioned that all EV-D68 cases had ‘moderate respiratory illness’. But the definition of moderate respiratory illness is unclear.

**Response**: We have revised this phrase to avoid confusion.  Accordingly the sentence now reads “All of 21 EV-D68 positive patients were aged less than 2 years, presented with a respiratory illness of short duration and none needed admission to intensive care units.”

 

- They collected samples from 2009 and 2015. But it is not mentioned in which year EV-D68 were detected.

**Response: **We have added details of the earliest detection of EV-D68 in our study.

# Peer review: Referee response for version 1

**DOI:** 10.21956/wellcomeopenres.12486.r24363

**Published:** 2017-07-31

**Authors:** Kristine M. Wylie

**Affiliations:** 1 Department of Pediatrics and The Genome Institute, Washington University School of Medicine, St. Louis, MO, USA

This is a nicely written report describing Enterovirus D68 prevalence and genome sequences in Vietnam. I have only minor comments.

1. The authors cite a real-time PCR assay that is specific for Enterovirus D68. I believe this paper cites the assay developed by the CDC. If so, the primary CDC reference should be cited. Does this assay detect viruses from all of the clades? If not or if it is unclear, this should be described as a limitation of the study.
2. Some additional details regarding the PCR assay and sequencing assays would be useful for reproducibility. Was there absolutely no deviation from the cited papers? What enzymes were used? RT-PCR instrument? How much material was used as input for the sequencing assays? What were the specific library construction methods?
3. In Figures 2 and 3, it is difficult to distinguish the dark and light blue colors. A color with more contrast should be selected for the Vietnam samples.
4. There seems to be a formatting issue. Sporadically lowercase "f" is replaced with an odd symbol in my copy of the paper. There are several examples in the second paragraph of the introduction, but this occurs elsewhere in the paper, too.

I have read this submission. I believe that I have an appropriate level of expertise to confirm that it is of an acceptable scientific standard.

# Response

**Published:** 2018-05-03

**Authors:** Nguyen Thi Han Ny

**Affiliations:** Oxford University Clinical Research Unit, Vietnam

This is a nicely written report describing Enterovirus D68 prevalence and genome sequences in Vietnam. I have only minor comments.

1. The authors cite a real-time PCR assay that is specific for Enterovirus D68. I believe this paper cites the assay developed by the CDC. If so, the primary CDC reference should be cited. Does this assay detect viruses from all of the clades? If not or if it is unclear, this should be described as a limitation of the study.

**Response**: We would like to thank you for your comments and suggestions.  In regards to the real-time method for EV-D68 detection we have added a reference to the original paper describing and evaluating the CDC method. As originally designed the assay should be able to detect viruses from Asia, the US and Europe. However the diagnostic utility of the assay has only been conducted in the US, and therefore some SE-Asian clades may have been missed. We added some sentences in the discussion to elaborate this limitation.

  

1. Some additional details regarding the PCR assay and sequencing assays would be useful for reproducibility. Was there absolutely no deviation from the cited papers? What enzymes were used? RT-PCR instrument? How much material was used as input for the sequencing assays? What were the specific library construction methods?

**Response**: Additional information about the PCR assay and whole genome sequencing approach has been added to the methods section.

  

1. In Figures 2 and 3, it is difficult to distinguish the dark and light blue colors. A color with more contrast should be selected for the Vietnam samples.

**Response**: Colours on the phylogenetic trees have been changed to show the Vietnamese isolates clearer.

  

1. There seems to be a formatting issue. Sporadically lowercase "f" is replaced with an odd symbol in my copy of the paper. There are several examples in the second paragraph of the introduction, but this occurs elsewhere in the paper, too.

**Response**: Please forgive our ignorance. But we have thoroughly checked the document on more than one computer and could not found any formatting issues.

# Peer review: Referee response for version 1

**DOI:** 10.21956/wellcomeopenres.12486.r24365

**Published:** 2017-07-25

**Authors:** Weihua Huang

**Affiliations:** 1 Department of Pathology, New York Medical College, Vahalla, NY, USA

As I read it, the manuscript needs certain revisions. For example, "Phylogenetic analysis indicated that the virus was imported into Viet Nam in 2008." should be changed to "Phylogenetic analysis suggested that the virus likely appeared in Viet Nam in 2008.". There were also typing errors, such as "Figure 1. Recombination analysis of EVD68-VN9", which should be "EVD68-VN5". In addition, the abbreviation should be given in its full name while it appeared at the first time.

 Some suggestions in revision:

1. Table 2 has little to demonstrate and thus could be deleted;
2. Phylogenetic tree on VP1 could be ignored, when more whole genome sequences are available;
3. The detail of isolates should be given in the phylogeny tree for readers to view, and the VN isolates could be colored differently to stand out;
4. With points 2) and 3) taken, the supplementary tables could be deleted;
5. More detail in method will be appreciated, such as sequencing depth, bootstrap numbers for BEAST, and so on.

After all, the manuscript should be more accurate in expression and concise in style.

I have read this submission. I believe that I have an appropriate level of expertise to confirm that it is of an acceptable scientific standard, however I have significant reservations, as outlined above.

# Response

**Published:** 2018-05-03

**Authors:** Nguyen Thi Han Ny

**Affiliations:** Oxford University Clinical Research Unit, Vietnam

As I read it, the manuscript needs certain revisions. For example, "Phylogenetic analysis indicated that the virus was imported into Viet Nam in 2008." should be changed to "Phylogenetic analysis suggested that the virus likely appeared in Viet Nam in 2008.". There were also typing errors, such as "Figure 1. Recombination analysis of EVD68-VN9", which should be "EVD68-VN5". In addition, the abbreviation should be given in its full name while it appeared at the first time.

**Response**: Thank you for your comments. We have revised the sentence speculating the timing of EV-D68 appearance in Vietnam as per the reviewer’s suggestion.

The document has been re-read and typing errors corrected and all abbreviations have been given their full name when being used for the first time.

**Some suggestions in revision:**

- Table 2 has little to demonstrate and thus could be deleted;

**Response**: We have removed table two as suggested.

- Phylogenetic tree on VP1 could be ignored, when more whole genome sequences are available;

**Response**: We think that presenting both the VP1 and the whole genome phylogenies would be informative for the readers. This is because using the VP1 tree gives a greater breadth of isolates in order to give global context of our isolates. Additionally, using both the VP1 tree and whole genome tree allows comparison of the two methods, with the whole genome confirming findings in the VP1

 

- The detail of isolates should be given in the phylogeny tree for readers to view, and the VN isolates could be colored differently to stand out;

**Response**: We have changed the colours of the phylogenetic tree and added details of the Vietnamese isolates to aid readers. As for the global sequences, adding the details (including sampling dates, locations and accession numbers) of all the sequences to the trees would result in rather busy phylogenetic trees.

 

- With points 2) and 3) taken, the supplementary tables could be deleted;

**Response**: Please refer to our previous response. In addition, as the reviewer appreciates these supplementary tables occupy little spaces online, while they provide enough details for reproducibility.

 

- More detail in method will be appreciated, such as sequencing depth, bootstrap numbers for BEAST, and so on.

**Response**: We have added additional information to the methods including details of laboratory processing and a more detailed description of the BEAST analysis.
